
JOURNAL INFORMATION
==============================
NLM Title Abbreviation: Atlanta Med Surg J
NLM Journal ID: 0001010

Atlanta Medical and Surgical Journal

Publisher: Biomedical Journal Digitization Project

ARTICLE INFORMATION
==============================
PMCID: PMC8853995
PMID: 35825843
Article version: 1
Subjects: Editorial and Miscellaneous

Dr. Storer’s Lectures

Print publication date: 1867 May
Volume: 8
Volume Series: New Ser
Issue: 3
First page: 144
Last page: 144
License: This work is free of known copyright restrictions. For more information, please see PMC Back Issue Digitization.
License URL: https://creativecommons.org/publicdomain/mark/1.0/

==============================

### DR. STORER'S LECTURES.

The Lectures proposed by Dr. H. R. Storer, of Boston, to be delivered during the first two weeks of June, we presume will be eminently interesting and instructive.

His connection with Dr. Simpson, of Edinburgh, as assistant in practice, and as one of the Editors of his Obstetrical Works, in 1854–5, beside other evidences of his familiarity with female diseases, give assurance that his course of Lectures on the Treatment of Surgical diseases of Women, will be practical and useful.

---

### TO PHYSICIANS.

At the request of several members of the profession,

### DR. HORATIO R. STORER

will deliver a private course of twelve lectures upon the Treatment of the

SURGICAL DISEASES OF WOMEN, during the first fortnight of June, at his rooms in Boston.

Gentlemen attending the course will be required to show their diplomas. Fee $50.

*Hotel Pelham, Boston, March* 29, 1867.
